# Phytochemical Characterization of Chinese Bayberry (*Myrica rubra* Sieb. et Zucc.) of 17 Cultivars and Their Antioxidant Properties

**DOI:** 10.3390/ijms160612467

**Published:** 2015-06-02

**Authors:** Xianan Zhang, Huizhong Huang, Qiaoli Zhang, Fangjuan Fan, Changjie Xu, Chongde Sun, Xian Li, Kunsong Chen

**Affiliations:** 1 Laboratory of Fruit Quality Biology, the State Agriculture Ministry Laboratory of Horticultural Plant Growth, Development and Quality Improvement, Zhejiang Provincial Key Laboratory of Horticultural Plant Integrative Biology, Zhejiang University, Hangzhou 310058, China; E-Mails: 11216049@zju.edu.cn (X.Z.); huizi1989519@hotmail.com (H.H.); zhangqiaoli0228@163.com (Q.Z.); cherish_nu@163.com (F.F.); chjxu@zju.edu.cn (C.X.); adesun2006@zju.edu.cn (C.S.); akun@zju.edu.cn (K.C.); 2 Department of Horticulture, Lishui Academy of Agricultural Sciences, Lishui 323000, China

**Keywords:** Chinese bayberry, fruit quality, phytochemicals, LC–ESI-MS/MS, antioxidant capacities

## Abstract

In order to fully understand the variations of fruit quality-related phytochemical composition in Chinese bayberry (*Myrica rubra* Sieb. et Zucc.), mature fruit of 17 cultivars from Zhejiang and Jiangsu provinces was used for the investigation of fruit quality attributes, including fruit color, soluble sugars, organic acids, total phenolics, flavonoids, antioxidant capacity, *etc*. Sucrose was the main soluble sugar, while citric acid was the main organic acid in bayberry fruit. The content of total phenolics and total flavonoids were positively correlated with 2,2-diphenyl-1-picrylhydrazyl (DPPH), ferric reducing antioxidant power (FRAP) antioxidant activity and 2,2ʹ-azino-bis(3-ethylbenzothiazoline-6-sulphonic acid) (ABTS) radical scavenging activity. Five anthocyanidins, *i.e*., delphinidin–hexoside (Dp–Hex), cyanidin-3*–O-*galactoside (C-3–Gal), cyanidin-3*–O-*glucoside (C-3–Glu), pelargonidin-3*–O-*glucoside (Pg-3–Glu) and peonidin-3*-O-*glucoside (Pn-3–Glu), and seven flavonols compounds, *i.e*., myricetin-3*-O-*rhamnoside (M-3–Rha), myricetin deoxyhexoside–gallate (M-DH–G), quercetin-3*-O-*galactoside (Q-3–Gal), quercetin-3*–O-*glucoside (Q-3–Glu), quercetin-3*–O-*rhamnoside (Q-3–Rha), kaempferol-3*–O-*galactoside (K-3–Gal) and kaempferol-3*–O-*glucoside (K-3–Glu), were identified and characterized among the cultivars. The significant differences in phytochemical compositions among cultivars reflect the diversity in bayberry germplasm, and cultivars of good flavor and/or rich in various health-promoting phytochemicals are good candidates for future genetic breeding of bayberry fruit of high quality. In conclusion, our results may provide important information for further breeding or industrial utilization of different bayberry resources.

## 1. Introduction

Chinese bayberry (*Myrica rubra* Sieb. et Zucc., Myricaceae) is a subtropical fruit tree native to China, and it has been cultivated for more than 2000 years in China. It is the most economically important plant in the Myricaceae family, and the bayberry fruit is quite popular due to its delicious taste and attractive color [1]. So far, there are more than 300 cultivars in China. Fruit ripens in late May and early July, depending on different cultivars and production areas, and Zhejiang and Jiangsu provinces are two main production areas in China, which account for about 45% of the annual total bayberry production on the market. The bayberry fruit is a rich source for natural phytochemicals, including soluble sugars, organic acids and phenolics [1,2,3,4]. Bayberry is also a fruit with high medicinal value and has diverse health-promoting properties [5]. The diverse bioactivities of bayberry pulp extracts include antioxidant [2,6,7], anti-inflammation [8], anti-cancer [9,10], anti-bacteria [11,12], anti-diarrhea [13] and anti-diabetes [14,15,16]. However, most of the current research only focused on 2–4 main cultivars on the market, and no more work has been carried out for the comprehensive investigation of the phytochemical compositions of bayberry germplasm.

The present study was designed to investigate the phytochemical compositions of fruit of 17 bayberry cultivars. High-performance liquid chromatography (HPLC) and liquid chromatography combined with electrospray ionization mass spectrometry (LC–ESI-MS/MS) were used for characterization and quantification of different phytochemicals. Bayberry cultivars with a high amount of health-promoting phytochemicals may have great nutritional potential. Such information may provide an important clue for further development and utilization of natural *M. rubra* resources.

## 2. Results and Discussion

### 2.1. Basic Quality Indexes of Different Bayberry Cultivars

Significant differences were found in fruit color, weight and total soluble solids (TSS, °Brix), among the cultivars tested (Table 1). Wandao (WD) showed the highest color index of red grapes (CIRG) of 12.39, while Shuijing (SJ) showed the lowest CIRG of 1.96. Wuzi (WZ) has the highest fruit weight (28.17 g), and it is more than three times that of Tanmei (TM) (8.81 g). TSS varied between 8.74 and 11.67 °Brix. Despite these differences, the edible rate of all of the fruit samples reached more than 94% (Table 1).

**Table 1:** Appearance and taste qualities of Chinese bayberry fruits of 17 cultivars. TSS, total soluble solids.

Cultivars (Abbreviation)	Color (CIRG)	Weight (g)	Edible Rate (%)	TSS (°Brix)	Fructose *	Glucose *	Sucrose *	Citric Acid *	Malic Acid *
Biqi (BQ)	8.83 ± 0.24	11.5 ± 0.53 ^fg^	95.39	9.51 ± 0.19 ^f^	11.55 ± 0.78 ^cdef^	9.64 ± 0.57 ^c^	54.36 ± 0.93 ^ef^	9.17 ± 0.2 ^g^	0.68 ± 0.01 ^f^
Ciji (CJ)	8.58 ± 0.15	14.02 ± 0.34 ^d^	96.5	11.41 ± 0.21 ^ab^	13.51 ± 1.22 ^ab^	11.26 ± 1.02 ^ab^	61.9 ± 1.24 ^b^	10.14 ± 0.09 ^f^	0.46 ± 0.04 ^ij^
Ding’ao (DA)	6.78 ± 0.18	13.00 ± 0.54 ^def^	96.46	8.89 ± 0.12 ^gh^	11.16 ± 0.54 ^ef^	9.17 ± 0.9 ^c^	43.87 ± 2.59 ^g^	7.84 ± 0.07 ^j^	1.04 ± 0.00 ^b^
Dongkui (DK)	7.51 ± 0.18	27.62 ± 0.86 ^a^	97.39	11.67 ± 0.17 ^a^	13.96 ± 0.54 ^ab^	11.96 ± 0.66 ^a^	65.44 ± 0.71 ^a^	10.51 ± 0.13 ^ef^	0.47 ± 0.03 ^hi^
Dayexidi (DYXD)	7.65 ± 0.10	13.68 ± 0.25 ^de^	97.08	11.55 ± 0.21 ^ab^	14.64 ± 1.07 ^a^	11.85 ± 0.72 ^a^	65.85 ± 1.38 ^a^	8.27 ± 0.1 ^hi^	1.16 ± 0.04 ^a^
Fenhong (FH)	4.41 ± 0.11	12.79 ± 0.52 ^def^	95.78	9.28 ± 0.1 ^fg^	10.84 ± 0.92 ^ef^	9.33 ± 0.45 ^c^	54.18 ± 2.32 ^ef^	9.49 ± 0.15 ^g^	0.39 ± 0.03 ^k^
Muyezhong (MY)	6.06 ± 0.13	10.65 ± 0.22 ^g^	94.84	10.74 ± 0.2 ^cde^	14.66 ± 0.32 ^a^	12.27 ± 0.97 ^a^	56.72 ± 2.42 ^cde^	12.72 ± 0.08 ^b^	0.50 ± 0.00 ^h^
Shenhongzhong (SH)	4.24 ± 0.11	13.74 ± 0.51 ^de^	96.07	9.68 ± 0.2 ^f^	12.49 ± 1.5 ^bcdef^	10.66 ± 0.31 ^abc^	57.72 ± 1.34 ^cde^	10.55 ± 0.15 ^e^	0.55 ± 0.01 ^g^
Shuijing (SJ)	1.96 ± 0.02	9.04 ± 0.37 ^h^	95.02	8.74 ± 0.22 ^h^	11.27 ± 0.91 ^def^	9.98 ± 0.65 ^bc^	40.41 ± 0.27 ^h^	22.06 ± 0.23 ^a^	0.12 ± 0.00 ^l^
Shuimei (SM)	5.92 ± 0.06	16.12 ± 0.4 ^c^	95.72	11.1 ± 0.21 ^bcd^	13.07 ± 1.5 ^abcd^	11.22 ± 1.32 ^ab^	56.15 ± 1.08 ^cde^	11.11 ± 0.21 ^c^	0.88 ± 0.03 ^c^
Tanmei (TM)	7.71 ± 0.67	8.81 ± 0.18 ^h^	96.37	10.48 ± 0.15 ^e^	13.34 ± 0.80 ^abc^	12.28 ± 1.33 ^a^	65.6 ± 2.44 ^a^	10.64 ± 0.29 ^de^	0.48 ± 0.00 ^hi^
Wandao (WD)	12.39 ± 0.24	12.39 ± 0.25 ^ef^	96.37	10.48 ± 0.15 ^e^	10.64 ± 0.93 ^f^	9.12 ± 0.58 ^c^	52.2 ± 3.34 ^f^	8.45 ± 0.06 ^h^	1.04 ± 0.01 ^b^
Wumei (WM)	6.87 ± 0.17	11.58 ± 0.91 ^fg^	94.3	11.24 ± 0.12 ^abc^	14.65 ± 0.17 ^a^	11.90 ± 0.52 ^a^	54.54 ± 2.23 ^ef^	10.5 ± 0.59 ^ef^	0.04 ± 0.00 ^m^
Wuzi (WZ)	6.32 ± 0.11	28.17 ± 0.97 ^a^	95.67	9.52 ± 0.21 ^f^	12.64 ± 0.96 ^bcde^	11.43 ± 0.82 ^ab^	55.52 ± 2.14 ^def^	10.94 ± 0.17 ^cd^	0.43 ± 0.01 ^j^
Xiaoyexidi (XYXD)	7.75 ± 0.12	11.82 ± 0.47 ^fg^	96.19	10.77 ± 0.21 ^cde^	13.46 ± 0.60 ^ab^	11.94 ± 0.96 ^a^	59.71 ± 1.04 ^bc^	8.05 ± 0.10 ^ij^	0.75 ± 0.00 ^d^
Zaodamei (ZDM)	6.40 ± 0.16	17.95 ± 0.42 ^b^	96.16	10.64 ± 0.20 ^de^	10.64 ± 1.78 ^f^	9.21 ± 1.28 ^c^	59.01 ± 3.44 ^bcd^	10.43 ± 0.06 ^ef^	0.71 ± 0.03 ^e^
Zaose (ZS)	5.39 ± 0.10	12.39 ± 0.29 ^ef^	96.69	9.65 ± 0.12 ^f^	13.56 ± 0.61 ^ab^	12.33 ± 0.62 ^a^	54.19 ± 1.68 ^ef^	9.53 ± 0.17 ^g^	1.05 ± 0.00 ^b^

* Results were the mean ± SD (*n* = 3) on a fresh weight (FW) (g) of bayberry pulp basis, and all of the soluble sugars and organic acids were quantified with their own standard curves (mg/g FW). Values within each column followed by different letters were significantly different at *p* < 0.05 according to Duncan’s new multiple range tests.

**Table 2:** Total phenolics, total flavonoids and antioxidant capacities of Chinese bayberry fruits of 17 cultivars.

Cultivars	Total Phenolics	Total Flavonoids	DPPH	FRAP	ABTS	APC Index *	Rank
BQ	2531.18 ± 72.20 ^a^	1911.35 ± 26.42 ^a^	3157.59 ± 174.53 ^ab^	3614.01 ± 28.39 ^a^	4507.55 ± 33.35 ^a^	97.9	2
CJ	2148.86 ± 35.28 ^c^	1483.14 ± 89.59 ^b^	2510.78 ± 55.7 ^def^	3019.99 ± 147.65 ^c^	2984.08 ± 14.51 ^d^	74.8	7
DA	1947.04 ± 55.07 ^e^	1263.80 ± 27.14 ^cd^	2205.37 ± 92.49 ^fg^	2729.98 ± 95.25 ^e^	3050.47 ± 193.62 ^d^	69.5	9
DK	1471.05 ± 20.71 ^i^	1051.65 ± 44.26 ^e^	1504.91 ± 90.28 ^hi^	1802.78 ± 14.45 ^h^	1946.99 ± 147.96 ^fg^	45.9	13
DYXD	1908.72 ± 24.82 ^ef^	1252.81 ± 65.42 ^cd^	2316.82 ± 183.46 ^efg^	2880.52 ± 43.98 ^d^	3008.36 ± 236.19 ^d^	71.7	8
FH	1325.83 ± 19.41 ^j^	1011.71 ± 39.35 ^e^	1305.07 ± 53.07 ^i^	1752.24 ± 55.23 ^h^	1673.71 ± 55.42 ^g^	41.5	16
MY	2306.26 ± 42.31 ^b^	1804.85 ± 86.09 ^a^	2619.48 ± 94.32 ^de^	3202.69 ± 56.49 ^b^	3504.95 ± 89.42 ^c^	81.4	4
SH	1349.16 ± 26.62 ^j^	1119.80 ± 105.65 ^de^	1311.04 ± 89.16 ^i^	1801.87 ± 41.82 ^h^	1847.15 ± 138.69 ^fg^	43.2	15
SJ	1312.20 ± 59.71 ^j^	845.34 ± 79.21 ^f^	1279.50 ± 15.01 ^i^	1720.86 ± 56.96 ^h^	1632.33 ± 66.52 ^g^	40.6	17
SM	1551.74 ± 41.60 ^h^	878.21 ± 70.59 ^f^	1762.73 ± 173.42 ^h^	2016.40 ± 69.88 ^g^	2171.34 ± 93.75 ^ef^	52.1	12
TM	2072.14 ± 39.68 ^cd^	1824.19 ± 5.74 ^a^	2950.79 ± 284.32 ^bc^	3065.64 ± 51.26 ^c^	3933.76 ± 198.22 ^b^	86.6	3
WD	2377.69 ± 45.35 ^b^	1598.33 ± 123.75 ^b^	3355.46 ± 158.57 ^a^	3602.48 ± 107.6 ^a^	4526.92 ± 223.96 ^a^	99.9	1
WM	1658.89 ± 82.16 ^g^	1288.49 ± 58.99 ^cd^	2316.17 ± 153.83 ^efg^	2376.05 ± 81.51 ^f^	2420.67 ± 167.94 ^e^	62.7	11
WZ	2039.46 ± 37.17 ^d^	1288.56 ± 41.21 ^c^	2736.93 ± 126.2 ^cd^	2712.93 ± 20.6 ^e^	3464.69 ± 214.17 ^c^	77.7	5
XYXD	1704.40 ± 53.35 ^g^	1249.37 ± 104.5 ^cd^	2069.13 ± 185.65 ^g^	2426.73 ± 23.1 ^f^	2772.39 ± 187.48 ^d^	63.4	10
ZDM	1836.98 ± 18.16 ^f^	1240.63 ± 42.51 ^cd^	2650.75 ± 240.6 ^cde^	2621.66 ± 56.55 ^e^	3462.83 ± 278.35 ^c^	76.0	6
ZS	1345.83 ± 29.15 ^j^	851.27 ± 6.41 ^f^	1394.91 ± 67.01 ^i^	1788.08 ± 69.42 ^h^	1902.78 ± 67.05 ^fg^	44.4	14

Results were the mean ± SD (*n* = 3) on a fresh weight (g) of bayberry pulp basis. Total phenolics were calculated as μg gallic acid equivalents (GAE)/g FW, and total flavonoids were calculated as μg rutin equivalents (RE)/g FW. Antioxidant capacities (DPPH, FRAP and ABTS) were calculated as μg trolox equivalent antioxidant capacity (TEAC)/g FW. Values within each column followed by different letters were significantly different at *p <* 0.05 according to Duncan’s new multiple range tests. * Antioxidant index score = [(sample score/best score) × 100], averaged for all three tests for each cultivar for the antioxiandant potency composite (APC) index.

### 2.2. Soluble Sugars and Organic Acids

Components and contents of individual sugars and organic acids were important factors determining fruit flavor. Further analysis showed that sucrose was the main soluble sugar that accounted for more than 60% of the total soluble sugars, while citric acid was the main organic acid that accounted for more than 80% of the total organic acids in all the bayberry cultivars tested (Table 1). The average sucrose content was 56.32 mg/g fresh weight (FW), and Dayexidi (DYXD) showed the highest sucrose contents (65.85 mg/g FW), while SJ showed the lowest sucrose content (40.41 mg/g FW). The average content of fructose and glucose were 12.71 and 10.91 mg/g FW, respectively. Among different soluble sugars, fructose has the highest sweetness, followed by sucrose. Beside the main cultivars, such as Biqi (BQ), Dongkui (DK) and WD, which contain relatively high content of sucrose and fructose, cultivars, such as Ciji (CJ), DYXD, Muyezhong (MY), TM and Xiaoyexidi (XYXD), also showed relatively high content of these two sugars. Therefore, they are good candidates for future genetic breeding of bayberry with good flavor.

For the organic acid composition, the average citric acid content was 10.6 mg/g FW, and the white cultivar SJ showed the highest citric acid content (22.06 mg/g FW), while the dark-red cultivar XYXD showed the lowest (8.05 mg/g FW) (Table 1). The average malic acid content was 0.63 mg/g FW, and it ranged from 0.04 mg/g FW in Wumei (WM) to 1.16 mg/g FW in DYXD. In addition, the sugars:acids ratio varied between 2.78 (SJ) and 9.79 (DYXD). Recently, by using RNA-Seq and bioinformatics, transcriptome analyses of gene expression were studied in relation to physiological and metabolic data associated with fruit taste during ripening of “Biqi” fruit in our group [17]. Unigenes involved in sucrose and citric acid metabolism were identified, where five sucrose phosphate synthase (*SPS*) genes and three glutamate decarboxylase (*GAD*) genes were characterized during fruit development and ripening [17].

### 2.3. Total Phenolics Total Flavonoids and Antioxidant Activities

The contents of total phenolics and total flavonoids in bayberry fruit extracts showed significant variation among the tested cultivars (*p* < 0.05) (Table 2). Total phenolics ranged from 1312.20 μg gallic acid equivalents (GAE)/g FW (SJ) to 2531.18 μg GAE/g FW (BQ) (Table 2). Total flavonoids ranged from 845.34 μg RE/g FW (SJ) to 1911.35 μg rutin equivalents (RE)/g FW (BQ) (Table 2). Such results were in the range of those previously reported for cultivars, such as SJ and BQ [2,18]. Since anthocyanins accounted for the major phenolics in the bayberry, cultivars with a dark color, such as BQ, TM and MY, showed relatively higher total phenolics and total flavonoid contents, while cultivars with a light color, such as SJ, Zaose (ZS) and Shuimei (SM) showed relatively lower total phenolics and total flavonoid contents.

The antioxidant activities of bayberry cultivars were evaluated by 2,2-diphenyl-1-picrylhydrazyl (DPPH), 2,2′-azino-bis(3-ethylbenzothiazoline-6-sulphonic acid) (ABTS) and ferric reducing antioxidant power (FRAP) methods. Generally speaking, cultivars with higher CIRG resulted in higher antioxidant activities, and three assays showed consistent results for the cultivars tested (Table 2). DPPH values varied from 1279.5–3355.46 μg trolox equivalent antioxidant capacity (TEAC)/g FW; FRAP values varied from 1720.86–3614.01 μg TEAC/g FW; and ABTS values varied from 1632.33–4526.92 μg TEAC/g FW among the 17 bayberry cultivars (Table 2). For a comprehensive comparison of the antioxidant capacities in bayberry of different cultivars, the antioxidant potency composite (APC) index was calculated according to the method described by Seeram *et al*. [19]. The APC index showed obvious variations ranging from 40.6 (SJ)–99.9 (WD) (Table 2). Correlation analysis showed that the overall APC was significantly positive correlated with the total phenolics (*r* = 0.969) and total flavonoids (*r* = 0.885), indicating that phenolic compounds were the main contributor of antioxidant capacity in bayberry. Similar results were shown in the study of bayberry pomace [7] and juice [20]. Recently, the hypoglycemic effect of bayberry fruit extract was reported, where antioxidants may play an important role, since bayberry fruit extract showed protective effects on pancreatic β cells against oxidative stress [14,15]. In another study, the inhibition activities of bayberry fruit extracts on the proliferation of SGC7901, AGS and BGC823 gastric cancer cells significantly correlated with their antioxidant activities [9]. Therefore, bayberry cultivars rich in phenolic compounds may result in high antioxidant activities, and they may have great health promoting potentials.

### 2.4. Identification and Quantification of Individual Phenolics

Individual phenolic compounds in bayberry fruit were further identified and quantified by HPLC-DAD and LC–ESI-MS/MS. Based on spectrum information, retention time of the standards, fragment ion information from LC–MS/MS or published literature, a total of 12 phenolic compounds, including five anthocyanins and seven flavonols, were identified (Table 3).

The anthocyanins identified were glycosides of delphinidin, cyanidin, pelargonidin and peonidin (Table 3). In bayberry fruit, cyanidin-3*–O-*glucoside (C-3–Glu) was the absolute predominant pigment compound, and it accounted for more than 68%–95% of total anthocyanins in the fruit extracts. Its contents showed significantly positive correlation with total phenolics (*r* = 0.883), total flavonoids (*r* = 0.808) and APC index (*r* = 0.896), indicating that C-3–Glu-rich cultivars might have great nutrition potential. Such results were consistent with previous reports [2,9,15,16,18]. In a study characterizing phenolic compounds in bayberry juice from 14 cultivars, C-3–Glu was the only anthocyanin detected, and its contents varied from undetectable to 514 mg/L [20]. In the present study, the C-3–Glu contents in the 17 cultivars tested varied from 9.34 (SJ)–912.24 μg/g FW (WD) (Table 4). Delphinidin–hexoside (Dp–Hex), cyanidin-3*–O-*galactoside (C-3–Gal), pelargonidin-3*–O-*glucoside (Pg-3–Glu) and peonidin-3*–O-*glucoside (Pn-3–Glu) were also identified and quantified (Table 3 and Table 4). Similarly, significant differences were found in the contents of these four anthocyanins, and SJ was the only cultivar for which no detectable anthocyanin was found besides C-3–Glu (Table 4). Recently, the anthocyanidin biosynthesis pathway, as well as transcriptional factors MYB, bHLH and WD40 were identified for the regulation of anthocyanin accumulation in bayberry [4,21].

The flavonols identified were glycosides of myricetin, quercetin and kaempferol. They included two myricetin glycosides, *i.e.*, myricetin-3*–O-*rhamnoside (M-3–Rha) and myricetin deoxyhexoside–gallate (M-DH–G), three quercetin glycosides, *i.e.*, quercetin-3*–O-*galactoside (Q-3–Gal), quercetin-3*–O-*glucoside (Q-3–Glu) and quercetin-3*–O-*rhamnoside (Q-3–Rha), and two kaempferol glycosides, *i.e.*, kaemferol-3*–O-*galactoside (K-3–Gal) and kaemferol-3*–O-*glucoside (K-3–Glu) (Table 3). On average, M-3–Rha and Q-3–Rha showed a higher amount than other flavonols, and both of them accounted for more than 50% of the total flavonols identified (Table 4). Significant differences in the contents of these flavonols were found among the cultivars. For example, among the three quercetin glycosides, Q-3–Gal contents varied from 0.16 (SJ)–74.47 μg/g FW (BQ), Q-3–Glu contents from 0.13 (SM)–9.11 μg/g FW (BQ) and Q-3–Rha contents from 3.31 (SM)–49.7 μg/g FW (BQ) (Table 5). Two kaempferol glycosides were in trace amounts, and K-3–Gal was undetectable in eight cultivars tested. The presence of myricetin, quercetin and kaempferol in the bayberry fresh, as well as after jam processing were previously reported by Amakura *et al*. [22]. After acid hydrolysis, quercetin was found as the dominant flavonol, followed by myricetin and kaempferol [22]. A similar observation was also found in the study of phenolic compounds in 14 bayberry juices; flavonol aglycons of myricetin, quercetin and kaempferol were tentatively identified and quantified after acid hydrolysis [6].

## 3. Experimental Section

### 3.1. Fruit Materials

Bayberry fruit at commercial maturity was used in the present study. Fruit of 17 cultivars was harvested from orchards of Zhejiang and Jiangsu provinces in June 2012 (Table 1), and fruit was transported to the laboratory of Zhejiang University, Hangzhou, within 6 h of harvest. Among them, Dayexidi (DYXD), Wumei (WM) and Xiaoyexidi (XYXD) were from Jiangsu provinces, while the rest of the 14 cultivars were from Zhejiang provinces. Uniform fruit free from blemishes and mechanical injury was selected for quality analysis. The whole fruit was frozen in liquid nitrogen, and the pulp was then separated and stored at −80 °C before analysis.

### 3.2. Chemicals and Reagents

All of the standards were of HPLC grade. Fructose, glucose, sucrose, citric acid, malic acid, C-3–Glu, M-3–Rha and Q-3–Glu were from Sigma–Aldrich (St. Louis, MO, USA). C-3–Gal, Pg-3–Glu and Pn-3–Glu were from Tokiwa phytochemical Co., Ltd. (Chiba, Japan). Q-3–Gal and Q-3–Rha were from J & K Scientific (Shanghai, China). K-3–Gal and K-3–Glu were from Tauto Biotech (Shanghai, China). Folin-Ciocalteu phenol reagent, gallic acid, 2,2-diphenyl-1-picrylhydrazyl (DPPH), (±)-6-hydroxy-2,5,7,8-tetramethylchromane-2-carboxylic acid (trolox) and acetonitrile of chromatographic grade were purchased from Sigma-Aldrich (St. Louis, MO, USA). Double-distilled water (ddH_2_O) was used in all experiments. All of the other solvents and reagents were of analytical grade and bought from Sinopharm Chemical Reagent Co., Ltd. (Shanghai, China).

### 3.3. General Analysis (Color, Fruit Weight, Edible Proportion and Total Soluble Sugar)

Fruit color measurement was carried out using the CIRG system according to previous reports with modification [3,23]. The raw data were obtained as L*, a* and b* by a MiniScan XE Plus Colorimeter (HunterLab, USA). Then, a* and b* were converted into hue angle (*H* = arctan (b*/a*)) and chroma (*C* = [(a*)^2^ + (b*)^2^]^0.5^), and CIRG was calculated as CIRG = (180 − *H*)/(L* + *C*). Two measurements were made for each fruit, and a mean value was obtained from the measurements of 15 fruits per cultivar. The weight of whole fruit and seed was measured from samples of 15 fruits per cultivar. The edible rate was calculated as the weight percentage of pulp to the whole fruit. TSS of 15 fruit per cultivar were determined with a Portable Brix Meter PR-101α (Atago, Japan) at 25 °C.

**Table 3:** Structural and chromatographic characteristics of investigated phenolic compounds. Dp, delphinidin; C, cyanidin; Pg, pelargonidin; Pn, peonidin; M, myricetin; Q, quercetin; K, kaempferol.

Chemical Structural	*T*_R_ (min)	λ_max_ (nm)	Molecular Weight	ESI-MS^2^ (m/s) *	Tentative Compounds	R_1_	R_2_	R_3_
Anthocyanins								
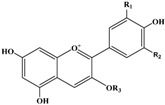	9.86	276, 520	465	465.2, 303.1	Dp-3–Hex	OH	OH	hexoside
	12.96	280, 515	449	449.0, 287.1	C-3–Gal	OH	H	galactoside
	14.91	280, 515	449	449.8, 287.1, 288.1	C-3–Glu	OH	H	glucoside
	17.87	278, 501	433	433.1, 271.1, 272.1	Pg-3–Glu	H	H	glucoside
	19.58	278, 515	463	463.0, 300.1, 301.1	Pn-3–Glu	OCH_3_	H	glucoside
Flavonols								
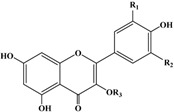	42.17	262, 349	464	463.1, 316.0, 317.0	M-3–Rha	OH	OH	rhamnoside
	42.94	256, 354	464	463.3, 301.1, 300.1	Q-3–Gal	OH	H	galactoside
	43.64	256, 353	464	463.3, 301.2, 300.1	Q-3–Glu	OH	H	glucoside
	47.55	265, 347	448	447.2, 285.0, 284.2	K-3–Gal	H	H	galactoside
	49.88	254, 349	448	447.2, 285.2, 284.1	K-3–Glu	H	H	glucoside
	50.55	256, 348	448	447.2, 301.2, 300.3	Q-3–Rha	OH	H	rhamnoside
	58.16	256, 349	616	615.1, 463.1, 317.0	M-DH–G	OH	OH	deoxyhexoside-gallate

* Anthocyanins were detected under +ESI mode; other flavonols were detected under −ESI mode.

**Table 4:** Anthocyanin contents (μg/g FW) in the pulp of the 17 Chinese bayberry cultivars.

Cultivars	Dp–Hex	C-3–Gal	C-3–Glu	Pg-3–Glu	Pn-3–Glu
BQ	11.10 ± 0.52 ^ab^	13.71 ± 1.32 ^cde^	837.32 ± 36.95 ^b^	10.38 ± 0.56 ^a^	19.09 ± 0.91 ^ab^
CJ	9.86 ± 0.50 ^cdefg^	11.35 ± 0.39 ^def^	636.49 ± 54.95 ^c^	9.32 ± 0.36 ^abcd^	15.04 ± 1.43 ^cd^
DA	11.46 ± 0.35 ^a^	14.26 ± 0.24 ^cd^	513.02 ± 8.18 ^e^	10.05 ± 0.52 ^ab^	15.17 ± 0.83 ^cd^
DK	9.37 ± 0.49 ^cdefg^	9.50 ± 0.52 ^g^	287.86 ± 9.68 ^g^	9.30 ± 0.52 ^abcd^	10.94 ± 0.65 ^fg^
DYXD	10.41 ± 0.39 ^abcd^	10.72 ± 0.53 ^fg^	901.43 ± 20.97 ^a^	9.64 ± 0.27 ^abcd^	17.17 ± 0.62 ^bc^
FH	8.69 ± 0.13 ^g^	8.75 ± 0.16 ^g^	74.55 ± 1.59 ^j^	8.65 ± 0.12 ^cd^	9.10 ± 0.16 ^g^
MY	10.22 ± 0.61 ^bcde^	10.34 ± 0.71 ^g^	582.63 ± 15.32 ^cd^	9.72 ± 0.59 ^abcd^	11.68 ± 1.79 ^ef^
SH	9.70 ± 0.67 ^cdefg^	9.70 ± 0.68 ^g^	143.71 ± 2.42 ^i^	9.56 ± 0.69 ^abcd^	10.70 ± 0.90 ^fg^
SJ	n.d.	n.d.	9.34 ± 0.14 ^k^	n.d.	n.d.
SM	9.05 ± 0.74 ^efg^	9.44 ± 0.82 ^g^	187.21 ± 5.27 ^hi^	9.47 ± 0.65 ^abcd^	9.69 ± 0.83 ^fg^
TM	10.67 ± 0.7 ^abc^	11.81 ± 0.22 ^def^	829.20 ± 42.32 ^b^	9.54 ± 0.52 ^abcd^	20.48 ± 1.46 ^a^
WD	9.60 ± 0.34 ^cdefg^	14.98 ± 1.80 ^c^	912.24 ± 84.84 ^a^	9.15 ± 0.53 ^bcd^	10.42 ± 0.78 ^fg^
WM	9.35 ± 0.18 ^defg^	10.70 ± 0.15 ^fg^	294.31 ± 7.35 ^g^	9.81 ± 0.21 ^abc^	9.95 ± 0.12 ^fg^
WZ	10.08 ± 0.48 ^bcdef^	35.35 ± 2.92 ^a^	537.98 ± 8.98 ^de^	9.78 ± 0.39 ^abc^	13.63 ± 1.34 ^de^
XYXD	8.95 ± 0.45 ^fg^	9.22 ± 0.41 ^g^	391.54 ± 6.95 ^f^	8.83 ± 0.35 ^cd^	10.30 ± 0.89 ^fg^
ZDM	8.67 ± 0.76 ^g^	23.20 ± 3.31 ^b^	340.02 ± 20.9 ^fg^	8.60 ± 0.69 ^d^	10.29 ± 1.31 ^fg^
ZS	9.20 ± 0.36 ^defg^	9.90 ± 0.38 ^g^	223.06 ± 8.71 ^h^	9.18 ± 0.23 ^bcd^	10.65 ± 0.62 ^fg^

n.d.—not detected. Results were the mean ± SD (*n* = 3) on a fresh weight (g) of bayberry pulp basis. C-3–Glu was quantified with its own standard curve, and the other four anthocyanins were quantified with C-3–G equivalents (μg/g FW). Values within each column followed by different letters were significantly different at *p <* 0.05 according to Duncan’s new multiple range tests.

**Table 5:** The flavonol contents (μg/g FW) in the pulp of the 17 Chinese bayberry cultivars.

Cultivars	M-3–Rha	M-DH–G	Q-3–Gal	Q-3–Glu	Q-3–Rha	K-3–Gal	K-3–Glu
BQ	50.33 ± 2.88 ^b^	1.87 ± 0.18 ^e^	74.47 ± 4.02 ^a^	9.11 ± 0.46 ^a^	49.70 ± 2.03 ^a^	4.26 ± 0.33 ^a^	4.32 ± 0.19 ^a^
CJ	18.38 ± 1.45 ^fg^	1.08 ± 0.10 ^f^	35.07 ± 1.07 ^c^	4.03 ± 0.23 ^d^	20.15 ± 1.77 ^c^	2.11 ± 0.05 ^c^	1.59 ± 0.07 ^d^
DA	61.50 ± 3.72 ^a^	1.21 ± 0.06 ^f^	0.19 ± 0.05 ^h^	3.53 ± 0.09 ^e^	51.73 ± 0.68 ^a^	n.d.	0.32 ± 0.05 ^g^
DK	28.09 ± 1.84 ^e^	1.79 ± 0.08 ^e^	0.26 ± 0.02 ^h^	0.85 ± 0.05 ^gh^	6.89 ± 0.3 ^g^	n.d.	0.69 ± 0.03 ^f^
DYXD	20.27 ± 0.33 ^f^	1.14 ± 0.09 ^f^	26.74 ± 0.45 ^d^	5.83 ± 0.19 ^b^	18.14 ± 0.29 ^c^	0.63 ± 0.03 ^e^	1.1 ± 0.04 ^e^
FH	14.49 ± 0.61 ^gh^	1.10 ± 0.08 ^f^	0.25 ± 0.04 ^h^	0.57 ± 0.05 ^h^	8.37 ± 0.32 ^fg^	n.d.	0.8 ± 0.02 ^f^
MY	40.40 ± 1.52 ^cd^	2.00 ± 0.19 ^e^	0.67 ± 0.14 ^h^	3.96 ± 0.25 ^d^	30.38 ± 0.75 ^b^	n.d.	1.09 ± 0.04 ^e^
SH	19.73 ± 0.63 ^f^	1.13 ± 0.07 ^f^	0.44 ± 0.04 ^h^	0.79 ± 0.05 ^gh^	11.18 ± 0.19 ^e^	n.d.	0.98 ± 0.00 ^e^
SJ	36.04 ± 1.33 ^d^	3.50 ± 0.18 ^c^	0.16 ± 0.01 ^h^	0.73 ± 0.09 ^gh^	6.94 ± 0.60 ^g^	n.d.	1.71 ± 0.06 ^d^
SM	39.15 ± 2.86 ^d^	1.70 ± 0.10 ^e^	0.21 ± 0.01 ^h^	0.98 ± 0.05 ^g^	10.36 ± 0.43 ^ef^	n.d.	0.71 ± 0.03 ^f^
TM	27.85 ± 1.47 ^e^	1.24 ± 0.08 ^f^	39.93 ± 1.71 ^b^	5.00 ± 0.1 ^c^	31.16 ± 2.26 ^b^	2.20 ± 0.08 ^bc^	2.42 ± 0.15 ^c^
WD	40.28 ± 3.64 ^cd^	4.37 ± 0.38 ^b^	0.97 ± 0.11 ^h^	3.46 ± 0.32 ^e^	18.52 ± 1.61 ^c^	1.19 ± 0.08 ^d^	1.65 ± 0.13 ^d^
WM	31.37 ± 1.91 ^e^	5.49 ± 0.15 ^a^	0.48 ± 0.02 ^h^	1.03 ± 0.08 ^g^	13.82 ± 0.43 ^d^	0.21 ± 0.01 ^f^	3.91 ± 0.07 ^b^
WZ	38.53 ± 1.55 ^d^	1.32 ± 0.09 ^f^	0.28 ± 0.01 ^h^	1.77 ± 0.08 ^f^	19.29 ± 0.68 ^c^	n.d.	0.26 ± 0.01 ^g^
XYXD	48.74 ± 0.28 ^b^	2.55 ± 0.15 ^d^	16.52 ± 0.29 ^e^	1.76 ± 0.13 ^f^	13.68 ± 0.21 ^d^	0.37 ± 0.04 ^f^	0.35 ± 0.04 ^g^
ZDM	44.10 ± 2.52 ^c^	0.96 ± 0.05 ^f^	11.69 ± 0.48 ^f^	1.53 ± 0.15 ^f^	14.82 ± 0.6 ^d^	1.34 ± 0.04 ^d^	0.36 ± 0.03 ^g^
ZS	10.73 ± 0.71 ^h^	4.41 ± 0.28 ^b^	5.89 ± 0.24 ^g^	0.13 ± 0.01 ^i^	3.31 ± 0.19 ^h^	2.35 ± 0.01 ^b^	0.25 ± 0.01 ^g^

n.d.—not detected. Results were the mean ± SD (*n* = 3) on a fresh weight (g) of bayberry pulp basis. The flavonols were quantified with their own standard curves, except M-DH–G, which was quantified with M-3–Rha equivalents (μg/g FW). Values within each column followed by different letters were significantly different at *p <* 0.05 according to Duncan’s new multiple range tests.

### 3.4. Quantification of Individual Sugars and Organic Acids

Soluble sugars and organic acids were prepared according to Zhang *et al*. [3]. Briefly, 2 g of frozen bayberry pulp was extracted in 5 mL of cold 80% ethanol and shaken for 10 min at 35 °C, followed by centrifugation at 4000 rpm for 5 min at room temperature. The residue was re-extracted twice following the same procedure. The supernatants of three extractions were pooled and made up to 25 mL with 80% ethanol. One milliliter of extract was sampled, and the solvent was removed in a centrifugal vacuum evaporator, then dissolved with 0.5 mL of ddH_2_O. All samples were filtered through a 0.22-μm membrane before HPLC analysis.

Individual sugars and organic acids were analyzed by HPLC assay (2695 pump; Waters). Individual sugars were separated in Ultimate^®^ XB-NH_2_ column (4.6 mm × 250 mm) at 25 °C, using acetonitrile/water (*v*/*v*, 80/20) as the mobile phase in a flow rate of 1 mL/min. The compounds were detected using a refractive index detector (Jasco, Japan). Individual organic acids were separated in an ODS C18 column (4.6 mm × 250 mm) at 25 °C, isocratically eluted with 50 mmol/L (NH_4_)_2_HPO_4_ buffer (pH = 2.7) as the mobile phase at a flow rate of 0.5 mL/min. The compounds were detected at 210 nm (2996 diode array detector; Waters). Quantification of individual sugars and organic acids was made by comparison with the peak areas of the standards.

### 3.5. Determination of Total Phenolics and Total Flavonoids

A fruit sample of 0.2 g was extracted using 1 mL methanol (with 1% formic acid) in 10-mL screw-top tubes. The extract was sonicated for 30 min, then centrifugation at 12,000× *g* for 10 min at room temperature. The extract was transferred into a new 10-mL tube, and the residue was extracted twice following the same procedure. The combined extracts were used for the analysis of phenolic compounds and antioxidant capacities.

Total phenolics of Chinese bayberry pulp of different cultivars was measured using a modified colorimetric Folin-Ciocalteu method [24]. Four milliliters of ddH_2_O and 0.5 mL of appropriately-diluted fruit extracts were placed in a test tube. Folin-Ciocalteu reagent (0.5 mol/L, 0.5 mL) was added to the solution and allowed to react for 3 min. The reaction was neutralized with 1 mL of saturated sodium carbonate. Absorbance at 760 nm was measured using a spectrophotometer (UV-2550, Shimadzu, Kyoto, Japan) after 2 h. Gallic acid was used as the standard, and data were expressed as μg GAE/g FW.

Total flavonoids of Chinese bayberry pulp of different cultivars was measured according to Jia, Tang and Wu [25] with some modification. One milliliter of ddH_2_O and 0.5 mL of appropriately-diluted fruit extracts were placed in a test tube. Sodium nitrite (5%, 75 µL) was added to the solution and allowed to react for 6 min before adding 150 µL of aluminum chloride (10%). After 5 min, 0.5 mL of sodium hydroxide (1 mol/L) were added. The final volume was adjusted to 2.5 mL with ddH_2_O. Absorbance at 510 nm was recorded immediately. Rutin was used as the standard and data were expressed as μg RE/g FW.

### 3.6. Antioxidant Capacity Assays

DPPH radical scavenging activity was measured according to Brand-Williams, Cuvelier and Berset [26] with modifications. The reaction for scavenging DPPH radical was carried out by adding 2 µL of sample to 198 µL 25 μg/mL DPPH solution at room temperature. Absorbance at 515 nm before (A_0_) and after (A_1_) the reaction (1 h) was recorded using a microplate reader (Synergy H1, Biotek, Winooski, VT, USA).

The FRAP assay was carried out according to Benzie and Strain [27] with modifications. Briefly, an aliquot of 100 µL properly-diluted fruit extracts was mixed with 900 µL of fresh FRAP working solution, which was prepared by mixing 100 mL acetate buffer (300 mmol/L, pH 3.6), 10 mL TPTZ solution (10 mmol/L in 40 mmol/L HCl) and 10 mL FeCl_3_ solution (20 mmol/L FeCl_3_ solution). Absorbance at 593 nm was recorded using a spectrophotometer.

ABTS radical scavenging ability was measured according to Re *et al*. [28] with modification. The ABTS solution was diluted with ethanol to an absorbance of 0.70 ± 0.02 at 734 nm on the day of analysis. An aliquot of 100 µL of properly-diluted fruit extracts was added to 3900 µL of the diluted ABTS solution, and the absorbance readings were taken after 6 min. Absorbance at 734 nm was recorded using a spectrophotometer.

For all three assays, trolox was used as the standard, and data were expressed as μg TEAC/g FW. All measurements were performed in triplicate. For each of the antioxidant method, an APC index was calculated according to the formula, antioxidant index score = [(sample score/best score) × 100] [19], and the APC index was calculated as the average of the antioxidant index score of each method.

### 3.7. HPLC-DAD and LC–ESI-MS/MS Analysis of Phenolic Compounds

Each individual phenolic compound in the fruit extracts was identified and quantified by LC–ESI-MS/MS and HPLC-DAD. Anthocyanins were detected at 520 nm and flavonols at 350 nm. Seven individual flavonoids, *i.e.*, C-3–Glu, M-3–Rha, Q-3–Gal, Q-3–Glu, Q-3–Rha, K-3–Gal and K-3–Glu, were quantified with their own standard curves according to the retention time and the chromatographic peak area in the HPLC system. Dp–Hex, C-3–Gal, Pn-3–Glu and Pg-3–Glu were quantified with C-3–Glu standard and expressed as C-3–Glu equivalent. M-DH–G was quantified as M-3–Rha equivalent. All tests were run in triplicate, and data were expressed as μg/g FW.

The flavonoid compounds were determined with an HPLC system (2695 pump, 2996 diode array detector, Waters) coupled with an ODS C18 analytical column (4.6 × 250 mm) [29]. The column was operated at a temperature of 25 °C. The compounds were detected between 200 and 600 nm. The mobile phase of HPLC consisted of 0.1% (*v*/*v*) formic acid in water (Eluent A) and of acetonitrile: 0.1% formic acid (1:1, *v*/*v*) (Eluent B). The gradient program was as follows: 0–40 min, 10%–38% of B; 40–60 min, 38%–48% of B; 60–70 min, 48%–100% of B; 70–75 min, 100%–10% of B; 75–80 min, 10% of B. Mass spectrometric analyses were performed by an Agilent 6460 triple quadrupole mass spectrometer equipped with an ESI source (Agilent Technologies, Santa Clara, CA, USA) that operated in the positive ionization and negative ionization mode. The nebulizer pressure was set to 45 psi, and the flow rate of drying gas was 5 L/min. The flow rate and temperature of the sheath gas were 11 L/min and 350 °C, respectively. Chromatographic separations were done under the same parameters as HPLC-DAD analysis on an X-Bridge C18 analytical column (4.6 × 250 mm) using an Agilent 1290 Infinity HPLC system (Agilent Technologies, USA). The eluent was split at 0.3 mL/min going through the mass detector. The Agilent Mass Hunter Workstation was used for data acquisition and processing.

### 3.8. Statistical Analysis

A completely randomized design was used in the present study. Experimental data were expressed as the mean ± standard deviation (SD). Data were analyzed using SPSS statistics Version 22 (SPSS Inc., Chicago, IL, USA) and plotted by Origin Pro 9.0. Significant differences among the samples were calculated using one-way ANOVA followed by Duncan’s new multiple range test at *p* < 0.05. Pearson correlation coefficients were calculated between antioxidant activity and phenolic contents at *p* < 0.05.

## 4. Conclusions 

Comprehensive evaluation and comparison of fruit quality-related phytochemical compositions of 17 Chinese bayberry cultivars were carried out in the present study. The genetic diversity of fruit color, taste and nutritional value was presented by a quality index, such as CIRG, soluble sugar, organic acid, phenolic content, flavonoid content, antioxidant capacities, *etc*. Twelve individual phenolic compounds were characterized by HPLC-DAD and LC–ESI-MS/MS. Besides the main cultivars on the market, cultivars, such as TM, WM, DYXD, MY and CJ, showed a relatively higher content of these sugars, while WD, CJ, MY and TM, showed a relatively higher content of phenolics and antioxidant capacities. Overall, variations in phytochemical compositions in bayberry fruit extracts reflect the diversity in bayberry germplasm, and these cultivars are good candidates for future genetic breeding of bayberry fruit of high quality.
